# Investigating COVID-19’s Impact on Mental Health: Trend and Thematic Analysis of Reddit Users’ Discourse

**DOI:** 10.2196/46867

**Published:** 2023-07-12

**Authors:** Jianfeng Zhu, Neha Yalamanchi, Ruoming Jin, Deric R Kenne, NhatHai Phan

**Affiliations:** 1 Department of Computer Science Kent State University Kent, OH United States; 2 Center for Public Policy and Health Kent State University Kent, OH United States; 3 College of Public Health Kent State University Kent, OH United States; 4 Data Science Department New Jersey Institute of Technology Newark, NJ United States

**Keywords:** COVID-19, Reddit, r/Depression, r/Anxiety, pandemic, mental health, trend analysis, thematic analysis, natural language processing (NLP), Word2Vec

## Abstract

**Background:**

The COVID-19 pandemic has resulted in heightened levels of depression, anxiety, and other mental health issues due to sudden changes in daily life, such as economic stress, social isolation, and educational irregularity. Accurately assessing emotional and behavioral changes in response to the pandemic can be challenging, but it is essential to understand the evolving emotions, themes, and discussions surrounding the impact of COVID-19 on mental health.

**Objective:**

This study aims to understand the evolving emotions and themes associated with the impact of COVID-19 on mental health support groups (eg, r/Depression and r/Anxiety) on Reddit (Reddit Inc) during the initial phase and after the peak of the pandemic using natural language processing techniques and statistical methods.

**Methods:**

This study used data from the r/Depression and r/Anxiety Reddit communities, which consisted of posts contributed by 351,409 distinct users over a period spanning from 2019 to 2022. Topic modeling and Word2Vec embedding models were used to identify key terms associated with the targeted themes within the data set. A range of trend and thematic analysis techniques, including time-to-event analysis, heat map analysis, factor analysis, regression analysis, and k-means clustering analysis, were used to analyze the data.

**Results:**

The time-to-event analysis revealed that the first 28 days following a major event could be considered a critical window for mental health concerns to become more prominent. The theme trend analysis revealed key themes such as economic stress, social stress, suicide, and substance use, with varying trends and impacts in each community. The factor analysis highlighted pandemic-related stress, economic concerns, and social factors as primary themes during the analyzed period. Regression analysis showed that economic stress consistently demonstrated the strongest association with the suicide theme, whereas the substance theme had a notable association in both data sets. Finally, the k-means clustering analysis showed that in r/Depression, the number of posts related to the “depression, anxiety, and medication” cluster decreased after 2020, whereas the “social relationships and friendship” cluster showed a steady decrease. In r/Anxiety, the “general anxiety and feelings of unease” cluster peaked in April 2020 and remained high, whereas the “physical symptoms of anxiety” cluster showed a slight increase.

**Conclusions:**

This study sheds light on the impact of COVID-19 on mental health and the related themes discussed in 2 web-based communities during the pandemic. The results offer valuable insights for developing targeted interventions and policies to support individuals and communities in similar crises.

## Introduction

### Background

The COVID-19 pandemic has had a profound impact on mental health, as individuals worldwide have been subjected to feelings of depression, anxiety, fear, guilt, and anger [1]. In the United States, a considerable increase in the symptoms of anxiety disorder and depressive disorder has been observed during the period from April to June 2020, compared with the same period in 2019 [2,3]. There were >310 million confirmed cases and 5.4 million deaths worldwide [4], underscoring the need to investigate the precise emotional and behavioral changes that have arisen in response to the pandemic. Multidisciplinary research is called for to address the psychological impact of quarantine, social isolation, and economic stress, among many other factors, on mental health [5].

Social media sites have become increasingly popular avenues for seeking and sharing health information, making them important tools for understanding the mental health impact of the pandemic. Especially, during the pandemic, social media use surged as billions of people stayed at home and practiced social distancing [6]. Platforms such as TikTok (ByteDance Ltd), Pinterest (Pinterest, Inc), and Reddit (Reddit Inc) reported growth in monthly active users in 2021 compared with 2019, with increases of 38%, 32%, and 30%, respectively [7]. Despite the increased use of social media during the pandemic, the relationship between social media posts and mental health during times of crisis is not yet fully comprehended.

### Prior Work

In recent years, natural language processing (NLP) and statistical techniques have been increasingly used to analyze social media posts, providing valuable insights into mental health issues. For example, Park et al [8] examined the thematic similarities and differences between and membership in 3 web-based mental health communities from Reddit using a text mining and visualization approach. The study used topic modeling to identify the most frequently discussed themes across the communities and explored the differences and similarities in the language used in each community [8]. Tadesse et al [9] developed a machine learning model using support vector machines to detect depression-related posts on Reddit. They extracted linguistic features such as negation, positive and negative sentiments, and medical terms from the text to classify the posts [9]. In a different approach, Kolliakou et al [10] used time-series regression analysis to investigate the relationship between mental health–related conversations on Twitter (Twitter, Inc) and the incidence of crisis episodes [10]. More recently, Liu et al [11] used time-to-event modeling to examine the transition patterns of other subreddits to r/SuicideWatch. They used Bayesian Poisson regression to analyze the temporal factors associated with the subreddit transitions. In addition, they used latent Dirichlet allocation (LDA) to identify key topics and examined the association between topic trends and subreddit transitions [11]. These studies demonstrate the potential of using social media data to identify individuals in need of support and gain a deeper understanding of the impact of public health crises on mental health.

Recent studies have tried to understand the impact of the COVID-19 pandemic on mental health, particularly on depression and anxiety, which have been identified as major consequences through social media content analysis. Thukral et al [12] used statistical and NLP methods, such as LDA topic modeling, to identify pandemic-related stress factors from Reddit posts, with young adults and students being particularly affected by stress related to academic and financial issues. Low et al [13] and Biester et al [14] highlighted the importance of social media platforms, for example, web-based communities such as r/Depression and r/Anxiety, as a source of data for studying the impact of the pandemic on mental health using NLP techniques. Marshall et al [15] implemented an NLP platform to analyze tweet frequency and identify prevalent discussion topics among United Kingdom residents, identifying consistent concerns such as the pandemic’s influence on mental health, lockdown-induced fear and anxiety, and anger and distrust directed at the government. Brewer et al [16] conducted a thematic analysis of discussion forum posts related to anxiety, depression, and obsessive-compulsive disorder (OCD) during the pandemic. The study demonstrated the potential of social media forums as a source of data for understanding the impact of public health crises on mental health [16]. These studies demonstrate the potential of NLP and social media data to provide valuable insights into mental health trends during the COVID-19 pandemic.

Despite previous research, measuring the emotional and behavioral changes in response to the pandemic remains challenging. A comprehensive understanding of the impact of the pandemic on population mental health through social media discourse is still lacking. Prior studies [12-16] mainly focused on relatively short periods during the early stages of the pandemic and lacked prepandemic baseline data and postpandemic mental health analysis. Moreover, few of these studies explored both the r/Depression and r/Anxiety subreddits, and none investigated the differences and relationships between them over time in terms of themes and factors. This study aimed to address these limitations by exploring the impact of the COVID-19 pandemic on mental health through a trend and thematic analysis of Reddit users’ discourse, specifically focusing on the r/Depression and r/Anxiety subreddits from 2019 to 2022. The analysis was conducted by examining users’ emotional expressions at 3 time points: before the pandemic, during key events, and after the peak of the pandemic. This study sought to investigate the evolution of mental health themes over time and answer the following research questions:

1. How did the discourse in mental health–related subreddit communities evolve between 2019 and 2021?
2. When did Reddit users start discussing COVID-19 within the r/Depression and r/Anxiety subreddits after the nationwide emergency declaration on March 13, 2020?
3. How do engagement patterns and the prevalence of the studied themes differ between the r/Depression and r/Anxiety communities over the course of the study period?
4. How did the COVID-19 pandemic impact the discussion of mental health topics on the r/Depression and r/Anxiety subreddits, as evidenced by the k-means clustering analysis?
5. How do the identified themes, such as economic stress and substance abuse, correlate with mental health outcomes such as suicidal ideation within the r/Depression and r/Anxiety subreddits?

In the *Results* and *Discussion* sections, we examine these 5 key questions that offer valuable insights into the pandemic’s effects on mental health, as revealed through the personal perspectives of the Reddit users engaging in conversations within the r/Depression and r/Anxiety communities. These 2 subreddits are among the largest web-based communities where people discuss and seek support for issues related to depression and anxiety, respectively, with >995,000 members in r/Depression and >609,000 members in r/Anxiety. These communities offer a vast amount of text data that can provide a unique perspective into the struggles experienced by users during the pandemic, covering the entire timeline from the prepandemic to postpandemic periods.

## Methods

### Data Collection

The Reddit platform allows users to post longer-form content and encourages discussion through comments. The demographics of the specific users of the subreddits examined in this study are not available; however, data on the general user base of the platforms have been provided. Approximately half of the Reddit user base is from the United States, with 22% of them being young adults aged 18 to 29 years and 14% of them being aged 30 to 49 years [17]. The platform witnessed an increase of 44% in daily active users in 2020, reaching 52 million.

The Pushshift multithread application programming interface (API) wrapper was used to download data from mental health–related subreddits, such as r/Addiction, r/ADHD (attention-deficit/hyperactivity disorder), r/Alcoholism, r/Anxiety, r/Autism, r/Bipolar, r/BPD (bipolar disorder), r/Bulimia, r/Depression, r/Drugs, r/HealthAnxiety, r/MentalHealth, r/OCD, r/PTSD (posttraumatic stress disorder), r/Schizophrenia, r/Selfharm, r/SocialAnxiety, and r/SuicideWatch. Details of the subreddits are provided in Tables S1 and S2 in Multimedia Appendix 1. We downloaded 3 million posts from 2019 to 2021. In addition, we acquired additional data sets for the r/Depression and r/Anxiety subreddits that extended through 2021 and 2022. The Pushshift multithread API wrapper is a tool for efficiently retrieving submissions from the Pushshift API using multithreading [18]. Multithreading is a programming technique where a single program or process can have multiple threads of execution running concurrently, each performing a different task.

### Data Preprocessing

In this study, we applied a series of cleaning steps to preprocess the posts. These techniques include expanding contractions, replacing nonalphanumeric characters with whitespace, converting text to lower case, replacing empty strings with not-a-number values, removing stopwords, and lemmatizing the text. Not-a-number is a special floating-point value that represents undefined or unrepresentable values. Stopwords are common words such as “a,” “an,” “the,” “and,” and “of” that are often removed from text data because they do not add much value to the analysis. Lemmatization is the process of reducing words to their base or dictionary form, which can improve the accuracy of text analysis by reducing the number of unique words. Proper handling of these techniques is crucial to avoid errors in data analysis. A detailed sample of the cleaned posts is provided in Table S3 in Multimedia Appendix 1.

### Extracting Terms Within the Target Theme

#### Overview

Understanding the prevalent themes in mental health–related subreddit posts during the pandemic can provide valuable insights into users’ attitudes, beliefs, and experiences related to mental health, as well as patterns and trends. Low et al [13] conducted a feature extraction from 15 subreddit posts and manually built lexicons about suicidality, economic stress, isolation, substance use, domestic stress, and guns. The terms associated with the themes are provided in Figure S1 in Multimedia Appendix 1. We used this as our baseline to construct the target themes for our study.

To verify the presence of these themes in our subreddit posts, we applied topic modeling to identify the 10 most discussed topics. In addition, we used the Word2Vec embedding model trained on the corpus of Reddit posts for the semantic refinement of theme terms.

#### Identifying Baseline Themes Using LDA Topic Modeling

Topic modeling with LDA is a powerful unsupervised machine learning technique used to discover hidden thematic structures within a large corpus of textual data. LDA assumes that documents are composed of a mixture of topics, and each topic is represented by a distribution of words. The *genism* library was used to perform LDA model estimation. LDA generates a predefined number of topics in posts across mental health subreddits, each characterized by a set of terms ranked by their probability of occurrence within that topic [19]. To analyze pandemic-related topics, a sample of 464,264 posts from 2020 to 2022 was taken from the r/Depression and r/Anxiety subreddits to feed into the LDA model for topic extraction.

#### Semantic Refinement of Theme Terms Using the Word2Vec Embedding Model

Word2Vec is a neural network–based algorithm used for NLP that efficiently processes large amounts of text data. One of the most intriguing and powerful features of Word2Vec is its ability to identify and manipulate semantic relationships between words. For example, a classic example for Word2Vec is “king−man+woman=queen.” This demonstrates the ability of the model to identify and manipulate semantic relationships between words, in this case, the gender of royalty. The model is able to learn that the vector distance between “king” and “man” is similar to that between “queen” and “woman,” allowing it to accurately predict the relationship between the 2 pairs of words. This type of analogical reasoning has been applied in various NLP tasks such as language translation, sentiment analysis, and text classification [20].

We used Word2Vec and the Embedding Projector Platform, both of which are available through TensorFlow (Google Brain Team) [21,22], to train and visualize semantic word relationships within 2 subreddit posts. The Embedding Projector Platform is a robust tool developed by Google’s TensorFlow team for exploring and visualizing high-dimensional data, such as word embeddings produced by the Word2Vec algorithm. Its user-friendly interface enables easy identification of related word clusters and semantic relationships and even the creation of custom embeddings by combining or modifying existing ones. Owing to its powerful visualization capabilities, the Embedding Projector Platform is now an indispensable tool for NLP and other high-dimensional data applications.

### Trend Analysis

Trend analysis is a widely used technique for identifying patterns and changes over time. It involves the analysis of data over a period of time to detect trends, such as increasing or decreasing values, changes in direction, or fluctuations in patterns. In our study, we applied several analytical methods, including time-to-event analysis, theme trend analysis, and k-means clustering analysis, to 2 subreddits from 2019 to 2022. These methods provided valuable insights into various aspects of the Reddit posts’ themes, such as COVID-19, economic, social, domestic, educational, substance, and suicide. We labeled each post with corresponding themes, such as labeling a post 1 for the economic feature if it contained any terms related to the economic theme and 0 otherwise. These labels were used as features in subsequent analyses. Examples of labeled samples can be seen in Figure S2 in Multimedia Appendix 1.

1. Time-to-event analysis, also known as survival analysis, is a statistical method used to analyze the time until an event of interest occurs. It is commonly used in medical research to analyze the time until a patient experiences a certain event, such as death or disease recurrence [23]. Time-to-event analysis was used to investigate the timing of the first post by unique authors containing COVID-19–related keywords after March 13, 2020, when the Trump Administration declared a nationwide emergency and issued an additional travel ban on non-US citizens traveling from 26 European countries owing to COVID-19 [24]. The 2020 data set was the primary focus, encompassing 10,852 unique authors in r/Depression and 6291 unique authors in r/Anxiety. Table S4 in Multimedia Appendix 1 provides samples of cleaned tokens of COVID-19 posts in both subreddits.
2. Theme trend analysis is a valuable tool that enables researchers to examine patterns and changes in data over time. It has wide-ranging applications, including social media research for mental health [25]. In our study, we used trend analysis to gain insights into public attitudes and identify emerging concerns during the pandemic by conducting a long-term analysis of the target themes that we previously defined, covering the period between 2019 and 2022.
3. K-means clustering is an unsupervised learning technique that groups similar documents together based on their content without the use of labeled data using the term frequency–inverse document frequency scheme to create vectors representing documents [26]. The elbow method was used to determine the optimal number of clusters. In this case, the technique was applied to the r/Depression and r/Anxiety subreddits to uncover latent topics in posts before, during, and after the pandemic.

### Thematic Analysis

Thematic analysis is a method of identifying and analyzing patterns, themes, and trends in qualitative data. In this study, we used various analytical methods, including heat map analysis, factor analysis, and ordinary least squares (OLS) regression, to gain insights into the relationships between the COVID-19, economic, social, domestic, educational, substance, and suicide themes in the r/Anxiety and r/Depression subreddits from 2019 to 2022.

1. The heat map analysis is a powerful visualization method used to display the distribution of selected themes in a large corpus of textual data. We used the heat map to represent the relationships between the themes in the 2 subreddits.
2. Factor analysis is a statistical method that identifies underlying patterns or structures within a data set by reducing the dimensionality. We performed factor analysis on 3 separate data sets: r/Depression, r/Anxiety, and a combined data set of both subreddits.
3. We applied the OLS regression method [27] to analyze the relationship between the “suicide” variable and the independent variables (COVID-19, economic, social, domestic, educational, and substance). This analysis helped us determine the influence of these factors on the occurrence of “suicide” and estimate the change in the dependent variable associated with a 1-unit change in each independent variable.

### Ethics Approval

To protect the privacy and confidentiality of the individuals whose data were analyzed, all study data were deidentified before analysis. The data analyzed in this study were obtained from publicly available sources and contain no identifiable information. The sample posts in this study were preprocessed by removing stopwords and lemmatizing, and the resulting tokens make it impossible to identify users’ information. No personal information, including author names or any other private information, was included in the data set. By addressing these ethical considerations, we conducted a valuable and trend study on the impacts of COVID-19 on the content posted by the r/Anxiety and r/Depression users. In addition, this research was partially supported by the National Science Foundation project (IIS-2041065), which was approved by the institutional review board at Kent State University under the reference number KSU IRB20-182. After this paper was accepted for publication, the Institutional Review Board at Kent State University was consulted regarding privacy ethics concerns and the study was deemed exempt (KSU 819).

## Results

### Trend Analysis

#### Overview

Trend analysis often involves the visualization of data on a graph or chart to identify patterns and trends. In this study, we used a variety of methods, including statistical analysis, time-series analysis, and k-means clustering analysis, to better understand the data and make informed decisions based on the insights gained. These methods enabled us to identify and analyze trends in the data, allowing us to gain a deeper understanding of the themes and patterns within the Reddit posts related to mental health during the pandemic***.***

#### Question 1: How Did the Discourse in Mental Health–Related Subreddit Communities Evolve Between 2019 and 2021?

**Figure 1:**
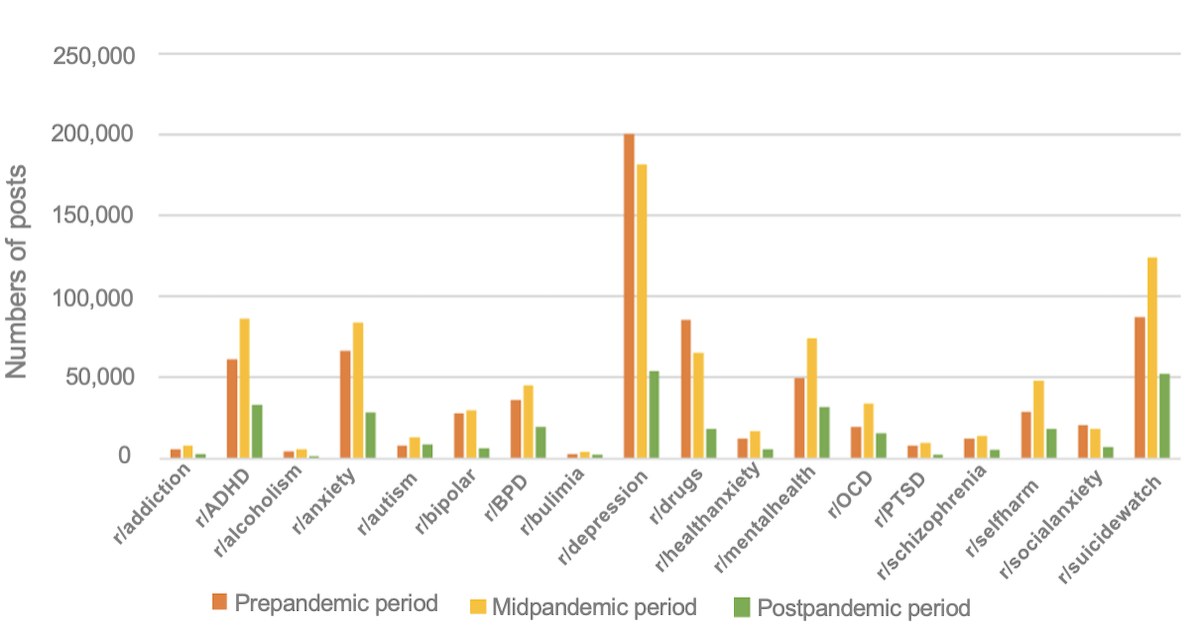
Mental health subreddit post distribution from 2019 to 2021. ADHD: attention-deficit/hyperactivity disorder; BPD: bipolar disorder; OCD: obsessive-compulsive disorder; PTSD: posttraumatic stress disorder.

In the initial stage of our analysis, we examined 18 mental health–related subreddits. Figure 1 illustrates a decline in the number of posts in the r/Depression subreddit and a rise in the number of posts in the r/Anxiety subreddit between 2019 and 2021.

The results indicate a decrease in the number of posts in the r/Depression subreddit during the midpandemic period and an increase in the number of posts in the r/Anxiety subreddit during the same period. A similar trend in tweets related to depression and anxiety can be found in Figure S3 in Multimedia Appendix 1.

Depression and anxiety are psychiatric disorders, often reflected in text written by undiagnosed individuals on social media. Medical experts can use linguistic markers to improve guidelines and treatments [28-30]. Focusing on the r/Depression and r/Anxiety subreddits allows us to study these specific disorders more closely, providing valuable insights into how individuals express their experiences and emotions during a global crisis such as the COVID-19 pandemic. In the r/Depression and r/Anxiety data sets, we extended the time frame to 2022, which was used in the following trend analysis and thematic analysis. The data set used in this study contains posts (652,452) collected from January 2019 to December 2022. It includes data obtained from the r/Depression and r/Anxiety subreddits. For the r/Depression subreddit, a total of 138,517 posts were collected in 2019, followed by 119,543 posts in 2020, 95,242 posts in 2021, and 85,283 posts in 2022, resulting in a cumulative total of 438,585 posts. Similarly, for the r/Anxiety subreddit, the data consisted of 49,295 posts in 2019, 54,053 posts in 2020, 53,992 posts in 2021, and 56,527 posts in 2022, accounting for a total of 213,867 posts. These statistics highlight the volume of posts obtained from each subreddit throughout the specified time period. The post distribution of the data set can be found in Figure S4 in Multimedia Appendix 1.

To validate the trend analysis on the Reddit data sets, we extracted tweets associated with anxiety and depression from our Twitter data set using hashtags and keywords. The trends observed in the number of posts on r/Depression and r/Anxiety were similar to those found in the number of tweets related to depression and anxiety hashtags and keywords. The hashtags and keywords used for filtering can be found in Table S5 in Multimedia Appendix 1.

#### Terms of Target Themes

Through LDA topic modeling, we extracted 10 topics based on the top 10 frequently occurring words, and Figure S5 in Multimedia Appendix 1 presents the keywords associated with each topic. These 10 topics were then used to verify the baseline themes, as shown in Figure S1 in Multimedia Appendix 1. Topic 9 focused on the economic theme, which included job search and work-related challenges. Social themes were found in topics 6 and 0, which covered personal relationships and social interactions and family and household matters, respectively. Domestic themes were mainly found in topic 0. Educational themes were highlighted in topic 8, including academic experiences and challenges. We excluded the gun theme because of a lack of relevant data and included the educational theme because of its high frequency. Although the COVID-19, substance, and suicide themes were not explicitly identified, they may be implicitly present within the broader categories of health and well-being, personal struggles, and life challenges during the pandemic. Our analysis identified 7 themes, namely economic, social, domestic, educational, substance, suicide, and COVID-19.

**Figure 2:**
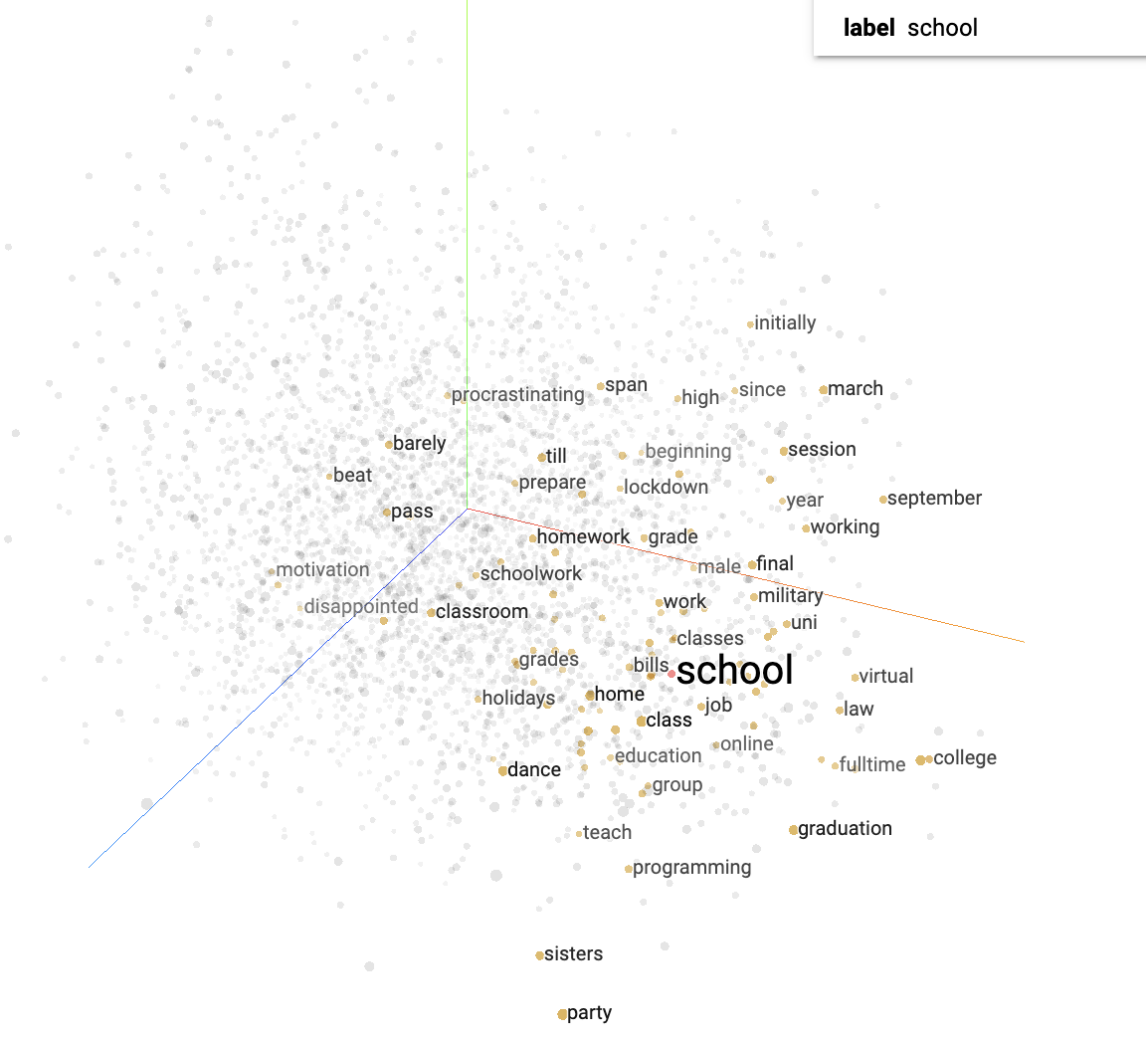
Visualization of the word “school” using the Embedding Projector Platform.

To preprocess the 652,452 corpora, we tokenized the posts from r/Depression and r/Anxiety between 2019 and 2022 and constructed a vocabulary. We generated training examples by creating pairs of target and context words within a defined window size of 2. We set the parameters of the model, such as dimension=128, batch size=1024, and buffer size=10,000, and trained the model using categorical cross-entropy loss and adaptive moment estimation as the optimization algorithm. Upon the completion of the training process with 20 epochs, the accuracy of the model was 0.633. The vectors and metadata files are available on GitHub [31], which can be loaded onto the Embedding Projector Platform [22] for projecting embeddings in 3D space and for the interactive exploration and analysis of semantic relationships among the corpora. Figure 2 displays the projecting visualization of the keyword “school” in the Embedding Projector Platform, illustrating the semantic relationship between “school” and the other words in the corpora in 3D space.

After considering the effects of the pandemic and reviewing the previous results, we manually constructed the final terms for the target themes. The resulting terms are presented in Table 1, with italicized words indicating extensions made using the top-ranked words from the Word2Vec pretraining model for the key terms.

**Table 1:** Terms of target themes.

Themes	Terms^a^
Economic	unemployed, economy, mortgage, *layoff*, *recession*, *stimulus*, evict, enough money, more money, pay the bill, owe, afford, wage, job, eviction, income, rent, *credit*, *salary*, *bills*, *laid*, *jobless*, *savings*, *lost job*, *fulltime*, *debt*, *financial*, *paycheck*
Social	*bullying*, *loneliness*, *emptiness*, quarantine, alone, lonely, lockdown, *distancing*, *insecurities*, no one cares, trapped, feel ignored, single, can’t see my, ignoring me
Domestic	domestic violence, abuse, yelling, fighting, single mom, single dad, single parent, hit me, slapped me, *divorced*, *abusive***,** *cheating***,** *separation***,** *toxic***,** *abused***,** *custody battles*
*Educational*	*exam*, *assignment*, *online classes*, *school closures*, *distance learning grade*, *homework*, *courses*, *school*, *presentation*, *classroom*, *test*, *virtual learning*, *hybrid learning*, *remote learning*, *online meeting*, *Zoom*, *Microsoft Teams*, *Google classroom*, *virtual classrooms*
Substance	smoke, *smoked*, drink, snort, *drugs*, *smoking*, alcohol, *nicotine*, *caffeine*, beer, substance, *ketamine*, *tablets***,** *valium***,** opioid, vodka, whiskey, whisky, meth, *addiction***,** *rehab***,** *relapse***,** *overdose*
Suicide	commit suicide, jump off a bridge, I want to overdose, will overdose, thinking about overdose, kill myself, killing myself, hang myself, hanging myself, cut myself, cutting myself, hurt myself, hurting myself, want to die, wanna die, do not wake up, do not want to be alive, wish it would all end, done with living, want it to end, all ends tonight, live anymore, living anymore, life anymore, be dead, end my life, death, *hopeless*, shoot me, kill me, suicide, no point, *intrusive*
COVID-19	corona, *coronavirus*, covid, *covid-19*, epidemic, infect, lockdown, pandemic, quarantine, viral, virus, mask**,** ventilator, symptomatic, incubation, transmission, immune, vaccine, *national emergency*, *flatten*

^a^Italicized words indicate extensions made using the top-ranked words from the Word2Vec pretraining model for the key terms.

#### Question 2: When Did Reddit Users Start Discussing COVID-19 Within the r/Depression and r/Anxiety Subreddits After the Nationwide Emergency Declaration on March 13, 2020?

**Figure 3:**
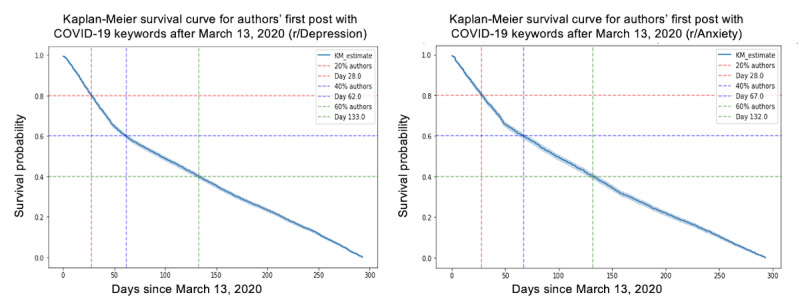
Kaplan-Meier survival curve in r/Depression and r/Anxiety. KM: Kaplan Meier.

We used time-to-event analysis to examine the initiation of COVID-19 discussions in the r/Depression and r/Anxiety subreddits following the nationwide emergency declaration on March 13, 2020. The Kaplan-Meier estimator [32], which is a nonparametric method for estimating the survival function, was applied to visualize the results. The Kaplan-Meier survival curve shows the estimated probability of not posting over time since March 13, 2020. Figure 3 illustrates the curve starting at 1.0 (ie, 100% probability of not posting) and decreasing over time as more authors post their first post with COVID-19 keywords in 2020. The vertical lines on the graph indicate the days on which a certain percentage of authors have posted, providing insights into the timing and probability of authors posting their first post after the national emergency declaration.

#### Question 3: How Do Engagement Patterns and the Prevalence of the Studied Themes Differ Between the r/Depression and r/Anxiety Communities Over the Course of the Study Period?

**Figure 4:**
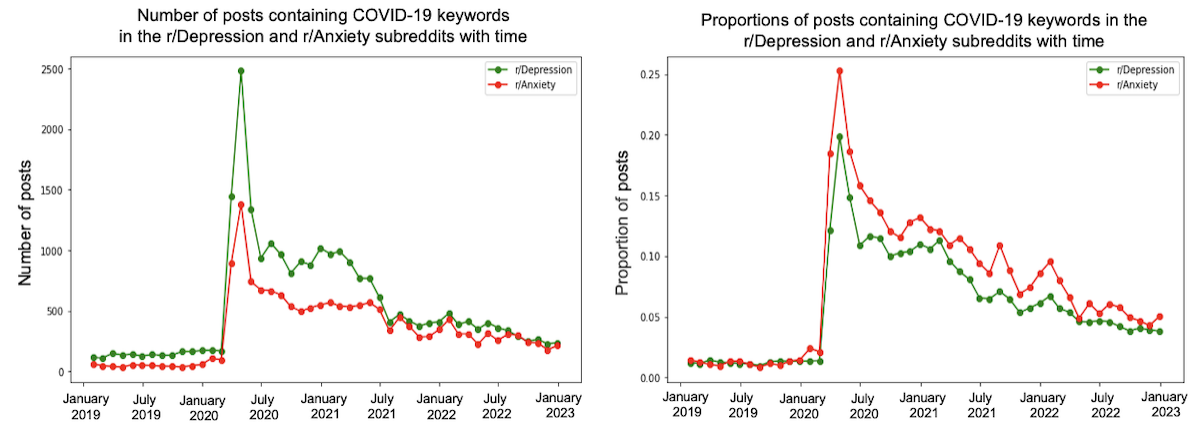
Number and proportion of posts that contain any keywords related to COVID-19.

**Figure 5:**
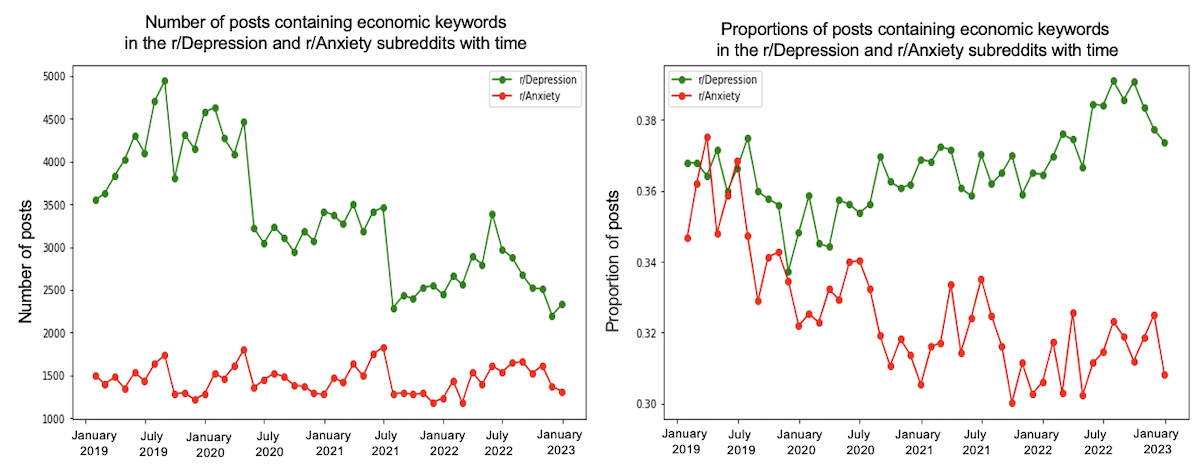
Number and proportion of posts that include any keywords related to the economic theme.

**Figure 6:**
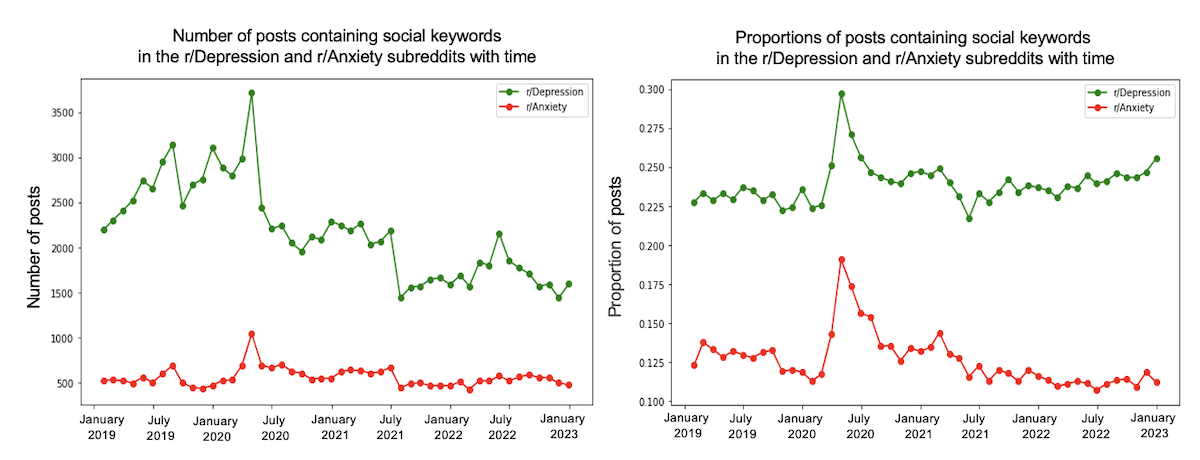
Number and proportion of posts that include any keywords related to the social theme.

**Figure 7:**
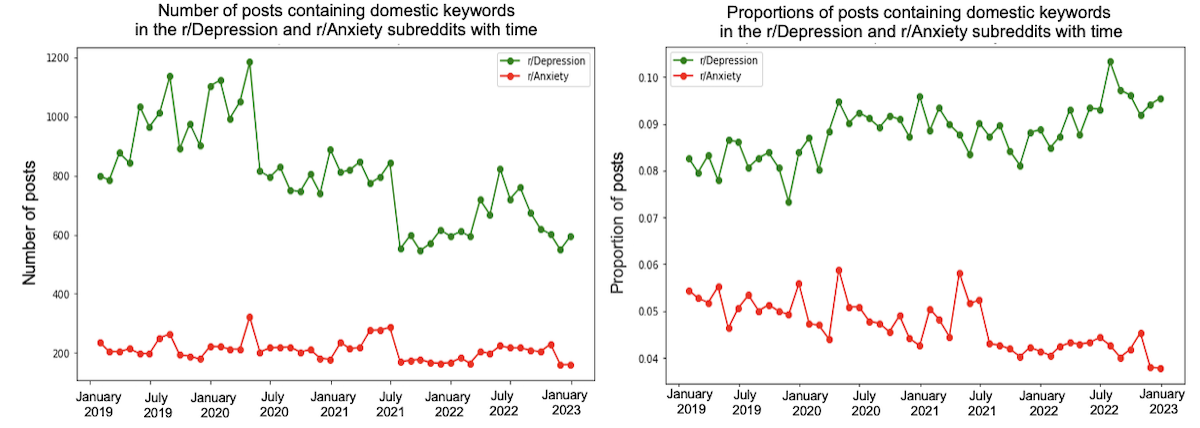
Number and proportion of posts that include any keywords related to the domestic theme.

**Figure 8:**
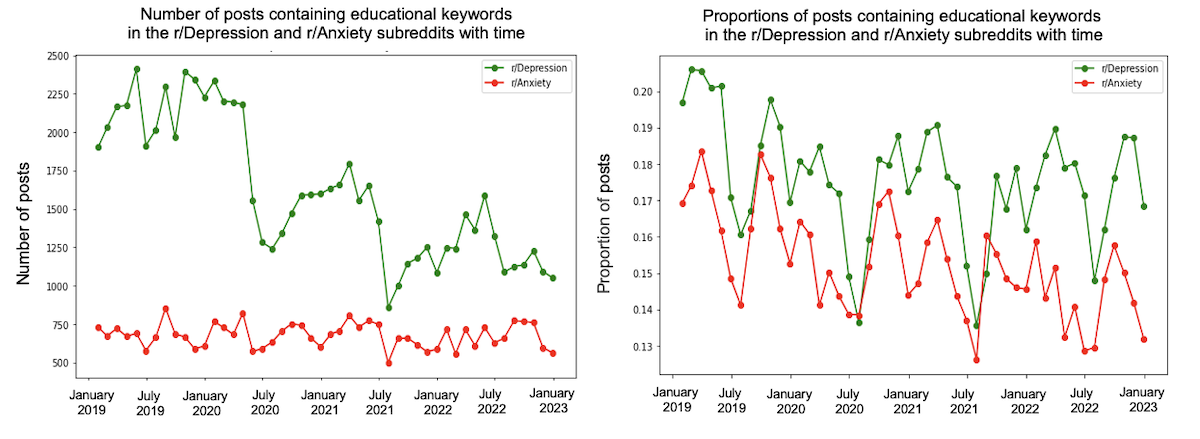
Number and proportion of posts that include any keywords related to the educational theme.

**Figure 9:**
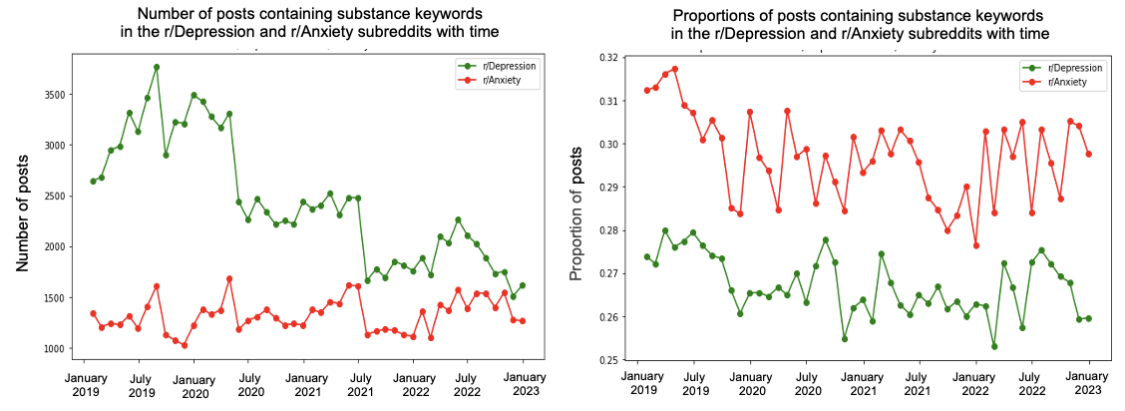
Number and proportion of posts that include any keywords related to the substance theme.

**Figure 10:**
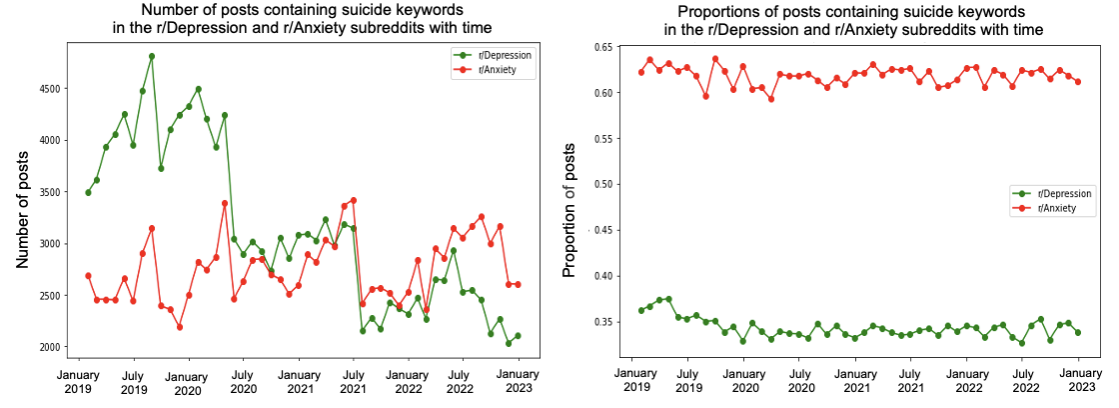
Number and proportion of posts that include any keywords related to the suicide theme.

We conducted a trend analysis to examine the differences in themes between the 2 subreddits over the specified period. Figures 4-10 depict the posting activity within the r/Depression and r/Anxiety communities on Reddit from 2019 to 2022, with a focus on the target themes COVID-19, economic, social, domestic, educational, substance, and suicide. The figure consists of 2 sections: the left side displays the distribution of the number of posts, whereas the right side shows the distribution of the proportion of posts for each community over time. The 2 lines in each section represent the r/Depression and r/Anxiety communities, providing insight into the varying dynamics of these web-based forums in the context of the specified themes.

#### Question 4: How Did the COVID-19 Pandemic Impact the Discussion of Mental Health Topics on the r/Depression and r/Anxiety Subreddits, as Evidenced by the K-Means Clustering Analysis?

**Figure 11:**
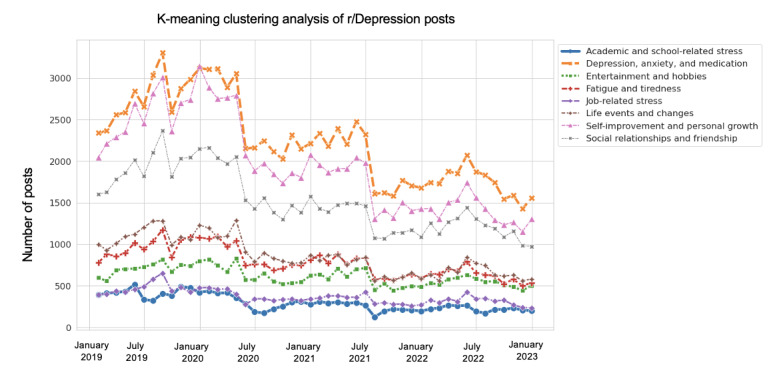
K-mean clustering analysis of r/Depression posts.

**Figure 12:**
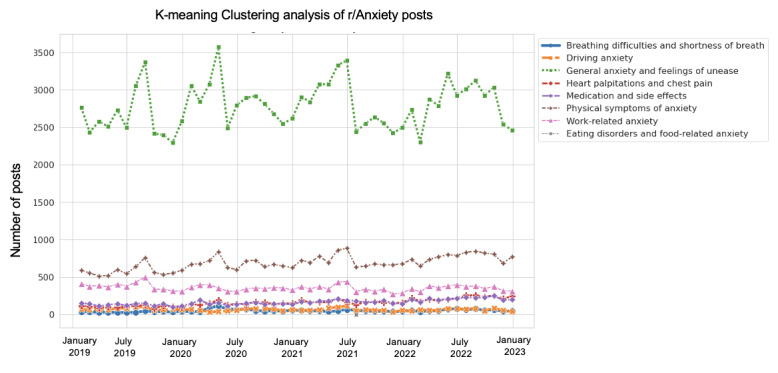
K-mean clustering analysis of r/Anxiety posts.

K-means clustering analysis was conducted on Reddit posts from the r/Depression and r/Anxiety subreddits with the aim of identifying distinct clusters of posts based on their textual content. The optimal elbow value for r/Depression was found to be 15, whereas the optimal elbow value for r/Anxiety was found to be 16. The results of k-means clustering are provided in Figures S6 and S7 in Multimedia Appendix 1. The clusters were then manually collated into 8 different latent clusters, which are provided in Table S6 in Multimedia Appendix 1, and the trends of these clusters from 2019 to 2022 were analyzed and displayed in Figures 11 and 12.

### Thematic Analysis

We used a combination of heat map analysis, factor analysis, and regression analysis to explore the correlation between the identified themes and mental health outcomes. Specifically, we aimed to address the following question.

#### Question 5: How Do the Identified Themes, Such as Economic Stress and Substance Abuse, Correlate With Mental Health Outcomes Such as Suicidal Ideation Within the r/Depression and r/Anxiety Subreddits?

##### Heat Map Analysis

**Figure 13:**
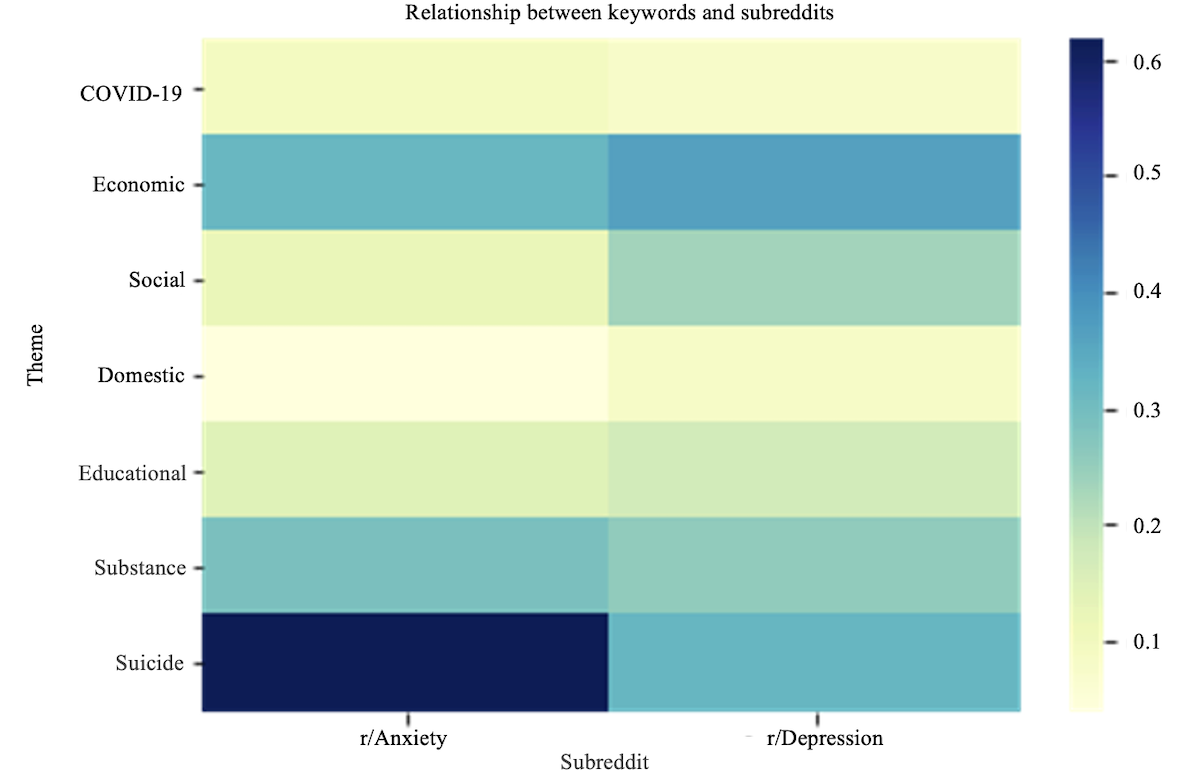
Heat map of the relationship between themes and subreddits.

We created a heat map to visually display the associations between the themes (COVID-19, economic, social, domestic, educational, substance, and suicide) and the 2 subreddits. A color gradient was used, where the deeper cell colors represent stronger associations. The x-axis of the heat map shows the 2 subreddits, with r/Anxiety on the left and r/Depression on the right, whereas the y-axis displays the selected themes. Each cell of the heat map represents the strength of the relationship between a given theme and subreddit. Figure 13 shows the resulting heat map.

##### Factor Analysis

**Figure 14:**
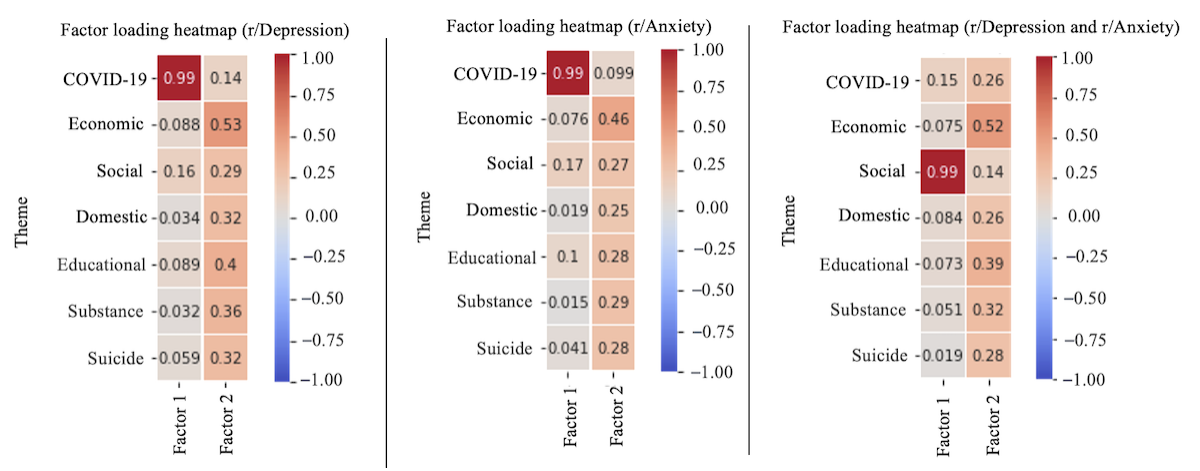
Factor loading heat map.

We used the Kaiser criterion (eigenvalues>1) to determine the optimal number of factors and applied the varimax rotation method to increase the interpretability of the results using the *Factor Analyzer* package in Python [33]. We calculated the eigenvalues of the correlation matrix for the 7 themes and plotted a scree plot to identify the “elbow” point, where the decrease in eigenvalues becomes less substantial. A scree plot is provided in Figure S7 in Multimedia Appendix 1. This helped us select the most meaningful structure in the data, with 2 factors identified as optimal for each data set. The factor loading matrix, shown in Figure 14, illustrates the strength and direction of the relationships between the themes and the 2 extracted factors, with higher absolute factor loadings indicating stronger relationships. Positive and negative loadings indicate direct and inverse relationships, respectively [34].

##### Regression Analysis

The OLS regression analysis on the r/Depression and r/Anxiety data sets from 2020 to 2022 showed that the suicide-dependent feature had a significant relationship with other independent features such as economic, social, domestic, educational, and substance. Figure S8 in Multimedia Appendix 1 presents the results, including the R-squared value, which indicates the proportion of variance in the dependent feature explained by the independent features (r/Depression: 0.055 and r/Anxiety: 0.031); the coefficients representing the strength and direction of the relationships; and the *P* values (*P*>|t|, both at .00) that determine statistical significance. In both subreddits, a *P* value <.05 indicates a statistically significant relationship.

## Discussion

We used a range of NLP techniques and statistical methods to perform a trend and thematic analysis, aimed at gauging the impact of COVID-19 on the mental well-being of individuals who are part of the r/Depression and r/Anxiety support groups. In the upcoming sections, we delve into each analysis in detail.

### Trend Analysis

#### Time-to-Event Analysis for Question 2

The results indicate that 20% of the authors posted by day 28 in both r/Depression and r/Anxiety. In addition, 40% of the authors posted by day 62 in r/Depression and day 67 in r/Anxiety, whereas 60% of the authors posted by day 133 in r/Depression and day 132 in r/Anxiety. The 28-day period after the announcement could be considered a critical window when mental health concerns started becoming more prominent for many individuals on Reddit.

This time-to-event analysis contributes to our understanding of how individuals affected by the pandemic increasingly turned to web platforms to discuss their mental health struggles. This emphasizes the need for mental health professionals and support organizations to recognize critical periods, such as the first 28 days following a major event, and prioritize resource allocation, interventions, and support measures accordingly. These findings can help inform future crisis management strategies to address the mental health impact of large-scale events on the general population.

#### Mental Health Theme Trend Analysis for Question 3

In early 2020, there was a noticeable rise in posts mentioning COVID-19 keywords (eg, corona, COVID, ventilator, vaccine, and mask) in both subreddits. A peak in April coincided with high COVID-19 cases and deaths in the United States, reflecting individuals sharing their fear and uncertainty.

Regarding the proportion of posts, the r/Anxiety community peaked at 0.25, whereas the r/Depression community reached 0.20 around April 2020. Subsequently, there was a sharp decrease until July 2020, followed by a stable decline. Throughout the pandemic, the r/Anxiety community maintained a slightly higher ratio than the r/Depression community, suggesting different levels of concern and coping mechanisms among web-based forums. However, this difference can be accounted for by many different factors, such as the variations in moderation principles and community guidelines. Therefore, it is not possible to draw any precise conclusions. This represents the limitation of the study that we attempt to address.

We observed prevalent themes such as economic stress, social isolation, domestic issues, education, substance use, and suicide ideation in the r/Depression and r/Anxiety subreddits. These themes presented varying trends and degrees of impact across the 2 communities, revealing the unique experiences and struggles faced by individuals coping with depression and anxiety during the pandemic.

In the r/Anxiety subreddit, the suicide theme emerged as the most dominant, accounting for around 63% of the posts, followed by economic stress and substance use. This indicates that the individuals in r/Anxiety may have experienced increased susceptibility to suicidal thoughts and harmful coping mechanisms during the pandemic. Conversely, in the r/Depression subreddit, economic stress was the most prevalent theme, emphasizing the significant impact of financial instability on individuals already grappling with depression. Social isolation was a shared concern across both communities, highlighting the negative impact of COVID-19 on interpersonal relationships.

The importance of addressing educational challenges during the pandemic was evident in both subreddits. The transition to remote learning and associated uncertainties exacerbated feelings of anxiety and depression among adolescents, with potential long-lasting consequences on their mental health.

By identifying and analyzing these important terms and themes, researchers, advocates, and practitioners can better understand the needs and experiences of individuals discussing mental health on social media during the pandemic. This can inform the development of more effective interventions and policies that are tailored to the unique challenges faced by individuals experiencing mental health concerns during the pandemic and beyond.

#### K-Means Clustering Analysis for Question 4

For the r/Depression subreddit, the number of posts related to the “depression, anxiety, and medication” cluster peaked in August 2019 and has been decreasing since 2020. The “self-improvement and personal growth” cluster showed a trend similar to that shown by the “depression, anxiety, and medication” cluster. The “social relationships and friendship” cluster showed a steady decrease since the beginning of the pandemic in 2020. The “fatigue and tiredness” cluster remained consistent throughout the period. Entertainment and hobbies did not show a significant increase or decrease after 2020. The “job-related stress” and “academic and school-related stress” clusters showed a stable trend throughout the period. The “life events and changes” cluster had a peak in April 2020 and has been decreasing since then. These findings in the depression group may suggest that the lockdown made people stay at home, where it is difficult to get any resources such as medication service, personal activities, and entertainment. This can be an explanation for the decrease of these clusters since 2020: because people did not have much chance to do these things, they did not discuss these things as much as they did in 2019.

Meanwhile, the r/Anxiety subreddit showed that general anxiety and feelings of unease peaked in April 2020 and remained at higher-than-average levels throughout the year. The “physical symptoms of anxiety” cluster remained stable but showed a slight increase since early 2020. The “work-related anxiety” cluster also remained relatively stable throughout the period. The “medication and side effects,” “heart palpitations and chest pain,” and “driving anxiety” clusters remained at consistent levels throughout the period. Overall, the pandemic seems to have had a significant impact on the clusters being discussed in the r/Anxiety subreddit, with many individuals seeking support for their general anxiety and unease during this challenging time.

### Thematic Analysis for Question 5

#### Heat Map Analysis

Through the heat map analysis, we were able to visually identify the patterns and relationships between the themes and subreddits. The heat map of the r/Anxiety subreddit revealed that the themes with the strongest relationship, in descending order, were suicide, economic, and substance. This suggests that individuals in this community were particularly affected by suicidal ideation, economic stress, and substance use during the analyzed period. The heat map of the r/Depression subreddit displayed the strongest relationships with the themes of economic, suicide, and social, suggesting that economic stress and social isolation were significant concerns for individuals in this community, along with ongoing struggles with suicidal thoughts.

#### Factor Analysis

The factor analysis of the r/Depression, r/Anxiety, and combined data sets from 2020 to 2022 offered crucial insights into the concerns and challenges faced by these web-based communities during this period. A discussion of the results highlights the following key findings:

1. COVID-19 as a prevalent concern: the strong association between the first factor and the “covid” theme (loading of 0.99) in both the r/Depression and r/Anxiety data sets suggests that the pandemic has had a significant impact on the mental health and well-being of the individuals in both communities. The results emphasize the need for mental health support and resources tailored to address pandemic-related stressors and anxieties.
2. Economic stress as a major issue: the second factor’s strong association with the “economic” theme (loadings of 0.53, 0.46, and 0.52) in all data sets indicates that economic stress has been a significant concern across both subreddits. This finding underscores the importance of addressing financial stress and providing support to those affected by job loss, reduced income, and other economic challenges.
3. Social concerns in the combined data set: the combined data set’s analysis revealed that social concerns (loading of 0.99) were also a primary theme, suggesting that interpersonal relationships and social interactions may be essential factors affecting mental health during this period. This observation highlights the need to consider social support and connectedness when addressing mental health issues within these communities.
4. Other themes with weaker associations: the remaining themes (“domestic,” “educational,” “substance,” and “suicide”) exhibited relatively weaker relationships with the extracted factors. However, their presence in the data sets suggests that they still hold relevance within these communities and should not be overlooked.

In conclusion, the factor analysis of the r/Depression, r/Anxiety, and combined data sets highlighted the importance of considering pandemic-related stress, economic concerns, and social factors when examining and addressing mental health issues during the period from 2020 to 2022. These findings contribute to our understanding of the challenges faced by these web-based communities and inform the development of targeted interventions and support.

#### Regression Analysis

Two OLS regression analyses were conducted on the r/Depression and r/Anxiety data sets to examine the relationship between suicide theme rates (dependent variable) and various independent variables, such as economic, social, domestic, educational, and substance themes. On the one hand, the r/Depression data set yielded an R-squared value of 0.055, indicating that the independent variables accounted for 5.5% of the variation in the suicide theme. On the other hand, the r/Anxiety data set showed an R-squared value of 0.031, explaining 3.1% of the variance in the suicide theme.

In the r/Depression data set, the coefficients revealed the following order of strength of association with the suicide theme: economic (0.1273), substance (0.0912), domestic (0.0815), educational (0.0745), and social (0.0773). All independent variables had statistically significant relationships with the suicide theme, as evidenced by their *P* values being <.05.

Similarly, in the r/Anxiety data set, the coefficients showed that the suicide theme had the strongest association with the economic theme (0.1093), followed by the substance (0.0890), social (0.0722), educational (0.0633), and domestic (0.0634) themes. All these relationships were statistically significant, with *P* values <.05.

In both data sets, the economic theme consistently demonstrated the strongest association with the suicide theme. This finding could indicate that financial stress and economic hardships may have a considerable impact on mental health, leading individuals to experience suicidal thoughts. In addition, the substance theme had a notable association in both data sets, suggesting a possible link between substance abuse and suicidal ideation.

### Limitations

We acknowledge certain limitations in our study. First, the data used for analysis were self-reported by users on subreddits, which may introduce social desirability biases. Second, the focus on r/Depression and r/Anxiety subreddits may not represent the mental health struggles of individuals across all web platforms or in real life, limiting the generalizability of our findings. Third, users may not necessarily discuss their own experiences when using the identified terms, and the identified theme terms may not encompass all terms related to the themes, which may lead to an overestimation of the relevance of certain themes. We also acknowledge that differences in post volumes could be attributed to various factors such as community guidelines and moderation principles. Finally, ethical concerns related to the use of social media data for mental health assessment, such as privacy and informed consent, need to be considered. Further research is needed to better understand the advantages and disadvantages of using social media for mental health assessment during the pandemic.

### Future Research

The study’s findings suggest that there are associations between certain themes, such as economic and substance use, and a higher proportion of suicide ideation. To gain a more comprehensive understanding of these associations, future research should explore them in greater detail and identify explanatory themes or factors that contribute to suicidal tendencies. Such research could help us develop more effective prevention and intervention strategies for individuals at risk of suicide. In addition, future studies should investigate other social media platforms and regions to enhance the generalizability of the findings. Future research could also examine how demographic factors, such as age, gender, and socioeconomic status, influence mental health discussions and emotions during the pandemic. By addressing these gaps, we can gain a more nuanced understanding of mental health impacts during global crises and develop targeted interventions and support systems for affected individuals.

### Conclusions

In conclusion, our study used a variety of NLP techniques to gain insights into the mental health struggles of the individuals participating in the r/Depression and r/Anxiety subreddits during the COVID-19 pandemic. Our time-to-event analysis revealed that the first 28 days following a major event could be considered a critical window for mental health concerns to become more prominent. The COVID-19 keyword trend analysis showed a peak in April 2020, reflecting individuals sharing their uncertainty during the pandemic. Our thematic analysis identified prevalent themes such as economic stress, social isolation, domestic issues, education, substance use, and suicide ideation, with varying trends and degrees of impact across the 2 communities. The factor analysis highlighted pandemic-related stress, economic concerns, and social factors as the primary themes affecting mental health during the analyzed period. The regression analysis showed that economic stress consistently demonstrated the strongest association with the suicide theme, whereas the substance theme had a notable association in both data sets. Finally, the k-means clustering analysis showed that the number of posts related to the “depression, anxiety, and medication” cluster decreased after 2020 in r/Depression, whereas the number of posts related to the “social relationships and friendship” cluster showed a steady decrease. In r/Anxiety, the “general anxiety and feelings of unease” cluster peaked in April 2020 and remained high, whereas the “physical symptoms of anxiety” cluster showed a slight increase.

However, we acknowledge certain limitations of our study, such as potential biases in the data collection and analysis methods, limited generalizability of the findings, and ethical concerns related to using social media data for mental health assessment. Further research is required to address these limitations and gain a more comprehensive understanding of mental health impacts during global crises. Future studies should explore the associations between themes in greater detail, investigate other social media platforms and regions, and examine the influence of demographic factors on mental health discussions and emotions during the pandemic. By addressing these gaps, we can develop more effective interventions and support systems tailored to the unique challenges faced by individuals experiencing mental health concerns during the pandemic and beyond.

## Appendix

Multimedia Appendix 1 Additional data sets details, sample Reddit posts, and analysis descriptions to support the main paper’s investigation into the impact of COVID-19 on mental health.

## Abbreviations

ADHD: attention-deficit/hyperactivity disorder
API: application programming interface
BPD: bipolar disorder
LDA: latent Dirichlet allocation
NLP: natural language processing
OCD: obsessive-compulsive disorder
OLS: ordinary least squares
PTSD: posttraumatic stress disorder

## Data Availability

The data and code used in this study are publicly available on GitHub [31].
