# Supplementary material for: Investigating COVID-19’s Impact on Mental Health: Trend and Thematic Analysis of Reddit Users’ Discourse
Source: J Med Internet Res. 2023 Jul 12;25:e46867. doi: 10.2196/46867 (PMC10365637; doi:10.2196/46867)
Supplement: Multimedia Appendix 1 [file jmir_v25i1e46867_app1.docx]

Investigating COVID-19’s Impact on Mental Health: Trend and Thematic Analysis of Reddit Users’ Discourse.

# 1 Data and Methods

## 1.1 Data collection

Data was downloaded from 18 subreddits from 2019 to the May 2021. The details of these subreddits can be found in Table S1.

Table S1 Popular Subreddits and Brief Description

| **SUBREDDIT** | **USERS** | **DESCRIPTION** |
| --- | --- | --- |
| r/ADHD | 1,213,846 | A place where people with ADHD and their loved ones can interact with each other exchanging stories, struggles, and non-medication strategies. Weekly threads to plan and notice the positive in our lives. |
| r/Anxiety | 463,187 | Discussion and support for sufferers and loved ones of any anxiety disorder. |
| r/Autism | 116,336 | Autism news, information and support. Please feel free to submit articles to enhance the knowledge, acceptance, understanding and research of Autism and ASD. |
| r/bipolar | 136,665 | A safe haven for bipolar related issues. We are a community  here not just a help page. Be a part of something that cares about who you are. |
| r/BPD | 137,284 | A place for those who have BPD (also known as EUPD), their family members and friends, and anyone else who is interested in learning more about the  mental illness. |
| r/Depression | 767,389 | Peer support for anyone struggling with a depressive disorder. |
| r/MentalHealth | 250,210 | The Mental Health subreddit is the central forum to discuss, vent, support and share information about mental health, illness and wellness. This sub is moderated by the South Asian Mental Health Alliance (SAMHAA), a non-profit society dedicated to mental health stigma reduction through skill development and community building. |
| r/OCD | 121,324 | A subreddit dedicated to discussion, articles, and images regarding OCD. Please read below for more information and resources about OCD and what this subreddit is! |
| r/SocialAnxiety | 301,269 | Feel nervous and/or shy in social situations? Whether it's approaching someone you're attracted to or if it's giving a presentation in class, everyone gets a little nervous at times. |

The following table S2 provides information on the number of raw data collected from

1st January 2019 to 31st May 2021, categorized into pre-pandemic, mid-pandemic, and post-pandemic periods.

Table S2 Raw Data Collection by Category and Period

| **Categories** | **From** | **To** | **Raw Data** |
| --- | --- | --- | --- |
| Pre-pandemic | 1st January 2019 | 31st December 2019 | 950,428 |
| Mid-pandemic | 1st January 2020 | 31st December 2020 | 1,139,632 |
| Post-pandemic | 1st January 2021 | 31st May 2021 | 424,517 |

We also acquired additional datasets for r/Depression and r/Anxiety subreddits that extended through 2021 and 2022. The number of posts in r/Depression and r/Anxiety subreddits from 2019 to 2022 were analyzed. The total number of posts for r/Depression over the four-year period was 438,585. For r/Anxiety, the total number of posts for the four-year period was 213,867.

## 1.2 Data processing

Extensive preprocessing was performed on each of the data files separately after extracting the data. These techniques include expanding contractions, replacing of non-alphanumeric characters with whitespace, converting text to lowercase, replacing empty strings with NaN values, removing stopwords, and lemmatizing the text. Table S3 illustrates an example of cleaned post.

Table S3 Example of cleaned post

| **Text** | **Cleaned text** |
| --- | --- |
| i dont get people sometimes. just some people have a tendency to make me upset/ angry/ or just straight up accept that me being alone is a good thing. | do not get people sometimes people tendency makes upset angry straight accept alone good thing |

## 1.3 Extracting terms within target themes

Low et al. (2020) conducted a feature extraction from fifteen subreddit posts, and manually built lexicons about suicidality, economic stress, isolation, substance use, domestic stress, and guns [13]. Figure S1 display the details of terms of themes.


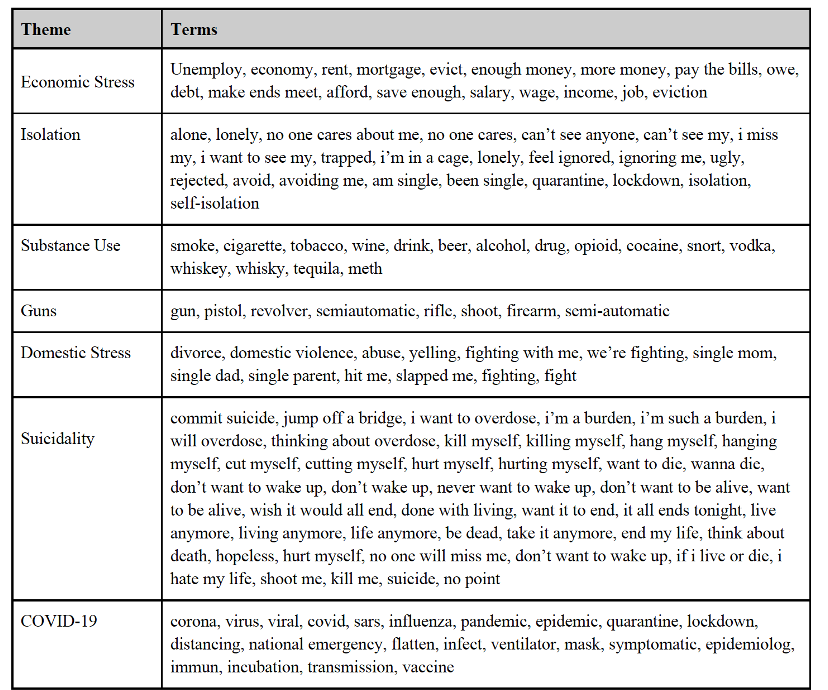


Figure S1. Tokens of themes in mental health Subreddits [1]

We labeled each post with corresponding themes, such as labeling a post 1 for the economic feature if it contained any terms related to the economic theme, and 0 otherwise. These labels were then used as features in subsequent analyses. Figure S2 display examples of labeled samples.


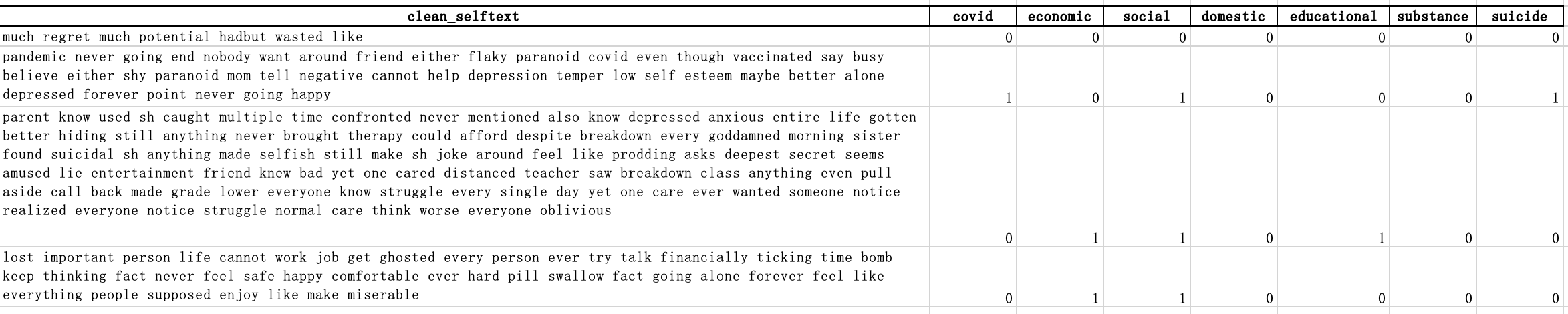


Figure S2. Sample of posts related 7 themes features

## 1.4 Longitudinal analysis

The 2020 dataset was the primary focus, encompassing 10,852 unique authors in r/Depression, and 6,291 unique authors in r/Anxiety. Table S4 displays samples of cleaned tokens of COVID-19 posts in both subreddit.

Table S4. Cleaned Tokens of COVID-19 Posts in r/Depression and r/Anxiety Subreddits

|  | **r/Depression** | **r/Anxiety** |
| --- | --- | --- |
| Sample Post1 | want **covid19** kill want virus obviously get hope **end** worst year life since 2016 | people **covid19** **scaring** crap much afraid getting virus scared people like wtf **worry** food house everyone going **quit job** hibernate |
| Sample  Post2 | first time **feeling** sad depressed going delayed school **school** asking **tuition fee** high to afford really squeezing dry also **covid** running money really wish end really tired done | worst thing ever entire family panicking **covid19** virus mom **lost job** except one barely make enough pay rent panicking everything |

# 2 Results

## 2.1 Overview of the Data

We analyzed a dataset of 262 million tweets spanning 33-weeks, which covers the initial spread of COVID-19 in the US starting from January 2020, and the implementation of stay-at-home orders and mandated quarantines in March 2020 to slow down the transmission rate.

Figure S3 illustrates a similar trend in depression and anxiety tweets, with a decrease in depression tweets but fluctuations observed, and an increase in anxiety tweets from February to April 2020 when COVID-19 policies, such as staying at home, were announced. The sudden changes and uncertainty in daily life caused increased panic, which may have led to the increase in anxiety-related tweets during this period.

| 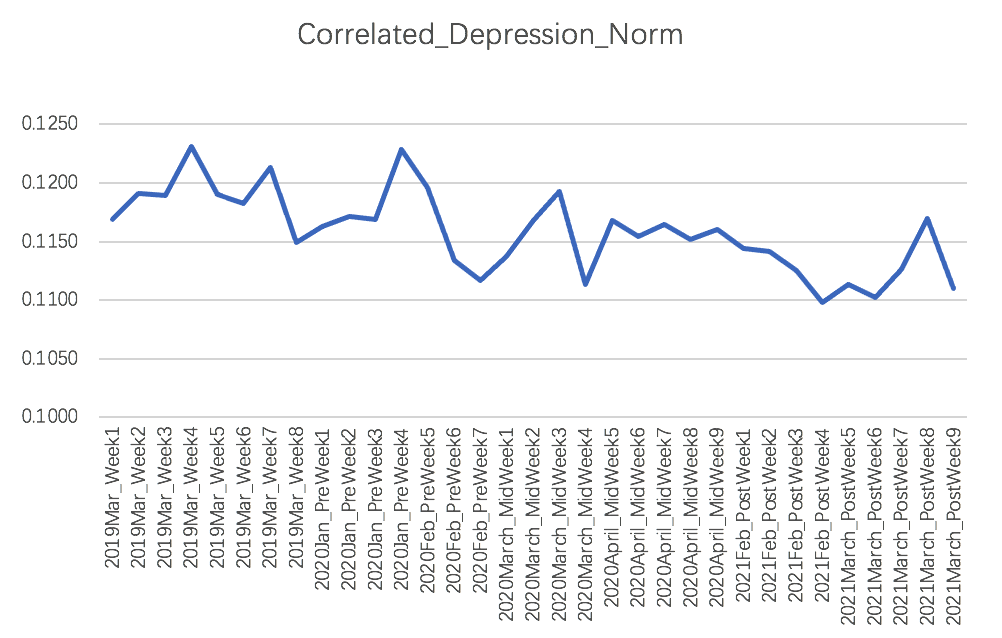 | 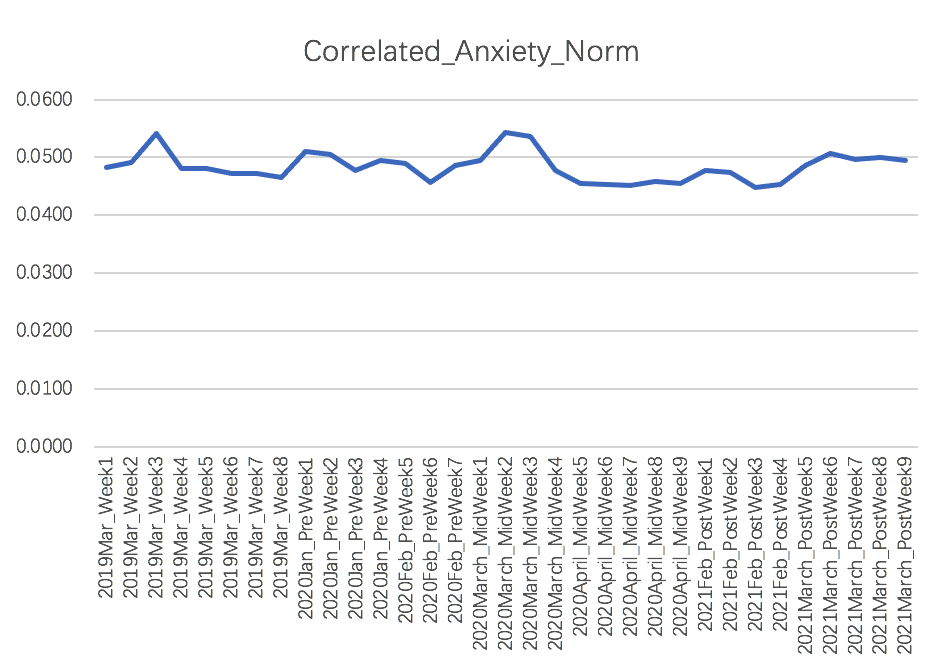 |
| --- | --- |

Figure S3. Normalized weekly Tweets Distribution for Depression and Anxiety.

Figure S4 shows the distribution of posts in r/Depression and r/Anxiety subreddits from 2019 to 2022. Over the four-year period, r/Depression had a total of 438,585 posts, with the number of posts decreasing from 138,517 in 2019 to 85,283 in 2022. In contrast, r/Anxiety had a total of 213,867 posts, with the number of posts increasing from 49,295 in 2019 to 56,527 in 2022.


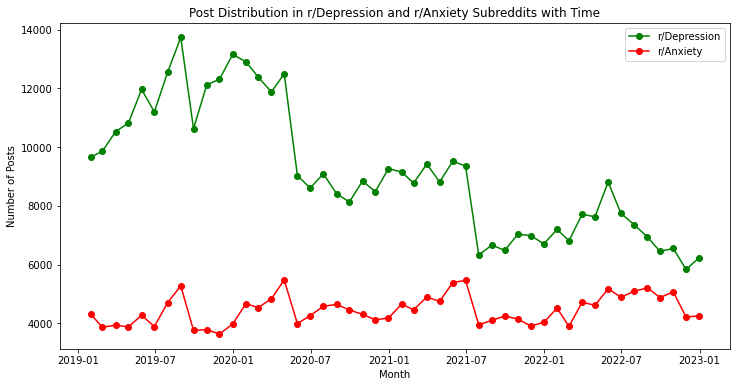


Figure S4. Post Distribution in r/Depression and r/Anxiety Subreddits (2019 to 2022)

To validate the trend analysis on Reddit datasets, we extracted tweets associated with anxiety and depression from our Twitter dataset using hashtags and keywords. Twitter data was obtained from the Internet Archive for the period of March 1^st^ to April 30^th^, 2020, and February 1^st^ to March 30^th^, 2021. We filtered 262 million tweets using the top 20 hashtags related to depression and anxiety. The hashtags and keywords used for filtering can be found in Table S5.

Table S5. Top 20 Hashtags for Depression and Anxiety on Twitter

|  | **Hashtags List** |
| --- | --- |
| Depression | #depression, #depressionquotes, #depressionawareness, #depressionhlp, #depressionsmenmes, #depressione, #depressionedits, #depressionrecovery, #depressionsucks, #depresionen, #depressionsupport, #depressionisreal, #depressionquote, #depressionkills, #depressionmeme, #depressions, #depressinandanxiety, #depressionhurts, #depressionedit, #depressionwarrior, #depressionglass |
| Anxiety | #anxiety, #anxietyrelief, #anxietyawareness, #anxietyhelp, #anxietysupport, #anxietyattack, #anxietydisorder, #anxietyproblems, #anxietyquotes, #anxietyquotes, #anxietywarrior, #anxietymemes, #anxietyfree, #anxietymanagement, #anxietyfighter, #anxietyisreal, #depressinandanxiety, #anxietyattacks, #anxietytips, #anxietyquote, #anxietycoach, #anxietydisorders |

## 2.2 Topic modeling

Topic modeling using Latent Dirichlet Allocation (LDA) is a powerful unsupervised machine learning technique used to discover hidden thematic structures within a large corpus of textual data. Figure S5 display the ten topics with their corresponding frequency terms.


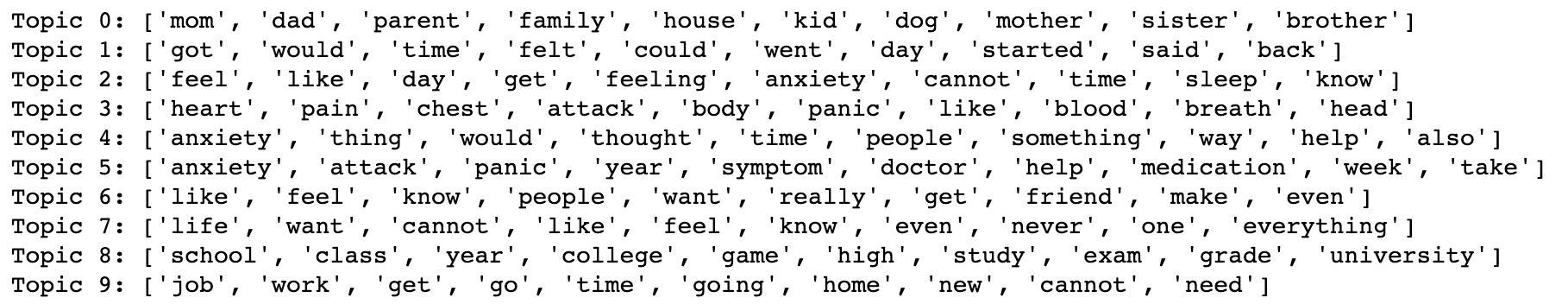


Figure S5. Top 10 terms of 10 topics of LDA

## 2.3 Longitudinal Analysis

### K-means Clustering Topic Trend Analysis

K-means clustering analysis was conducted on Reddit posts from the r/Depression and r/Anxiety subreddits, with the aim of identifying distinct clusters of posts based on their textual content. The optimal elbow value for r/Depression was found to be 15, while for r/Anxiety it was 16. Figure S6 and Figure S7 shows the result of K-means clustering for both subreddits.


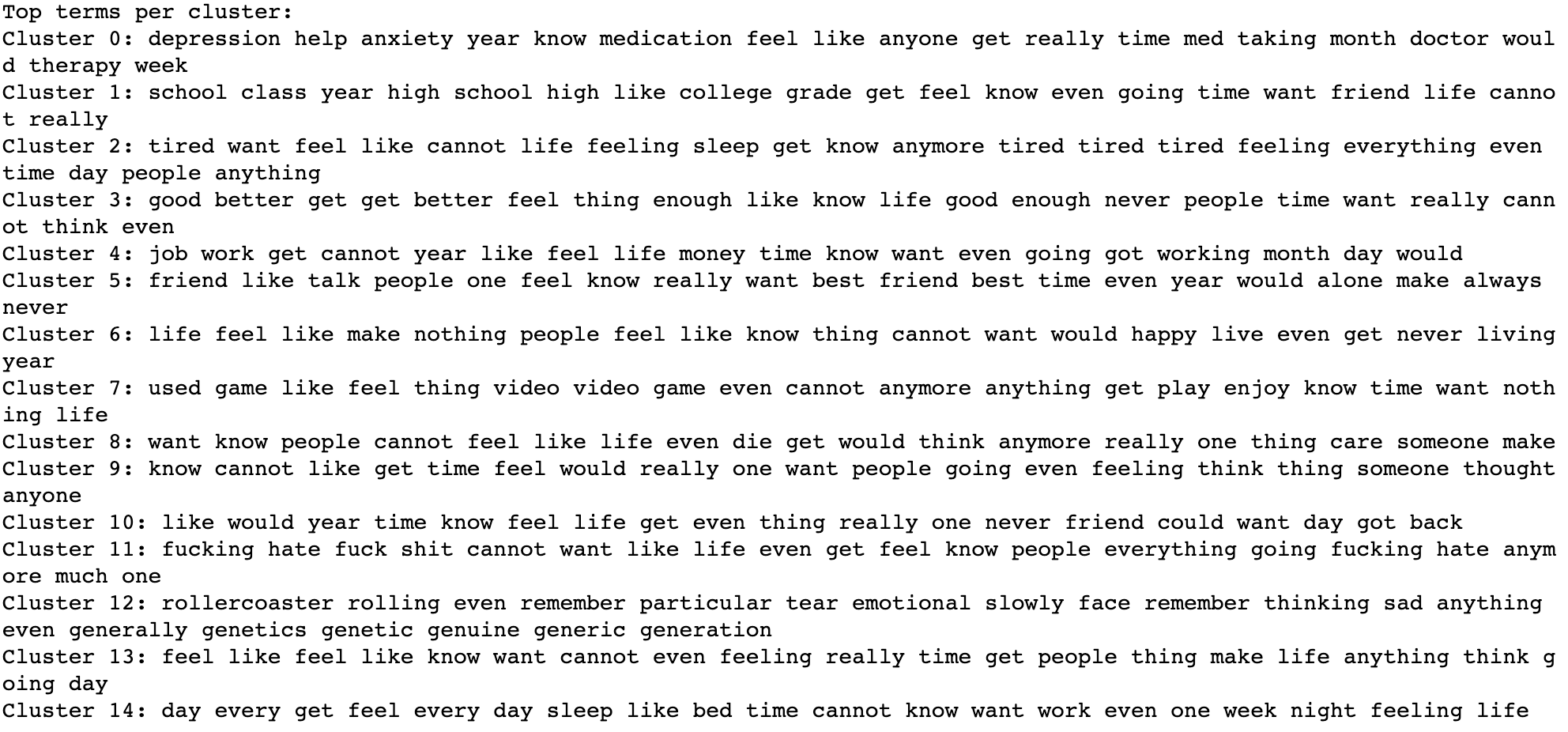


Figure S6. Top terms of clustering for the r/Depression


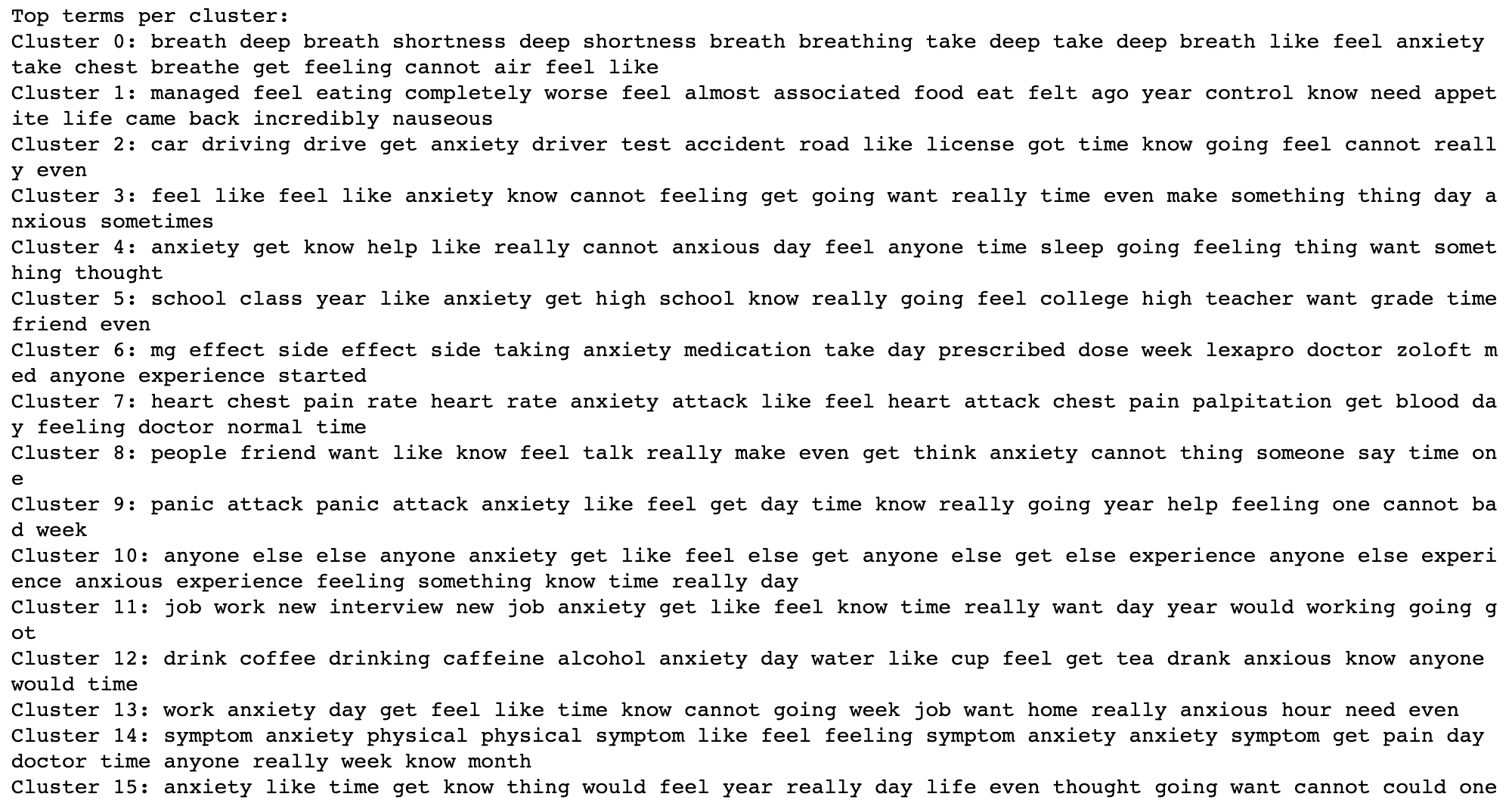


Figure S7. Top terms of clustering for the r/Anxiety

The clusters were then manually collated into eight different latent clusters, Table S6 displays the latent clusters in both subreddits and their corresponding number of posts in each subreddit.

Table S6. Results of Collated clusters

| **r/Depression** | | **r/Anxiety** | |
| --- | --- | --- | --- |
| Collated Cluster | Number of Posts | Collated Cluster | Number of Posts |
| Depression, anxiety, and medication | 107490 | General anxiety and feelings of unease | 133611 |
| Self-improvement and personal growth | 94056 | Physical symptoms of anxiety | 32915 |
| Social relationships and friendship | 72792 | Work-related anxiety | 17170 |
| Life events and changes | 41062 | Medication and side effects | 7880 |
| Fatigue and tiredness | 37770 | Heart palpitations and chest pain | 7645 |
| Entertainment and hobbies | 29452 | Driving anxiety | 3152 |
| Job-related stress | 17770 | Breathing difficulties and shortness of breath | 2371 |
| Academic and school-related stress | 14187 | Eating disorders and food-related anxiety | 1123 |

## 2.3 Thematic Analysis

#### Factor Analysis

Factor analysis is a statistical method that identifies underlying patterns or structures within a dataset by reducing its dimensionality. The objective is to group the given variables ('covid', 'economic', 'social', 'domestic', 'educational', 'substance', and 'suicide') into fewer factors that encapsulate the most significant patterns in the data.

To determine the optimal number of factors using the eigenvalue criterion (Kaiser criterion), first, calculate the eigenvalues of the correlation matrix of the given variables, see Figure S7.

| 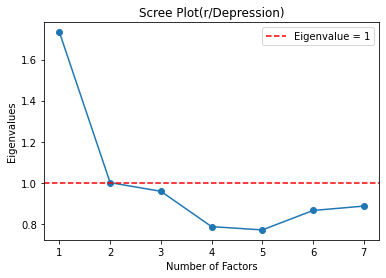 | 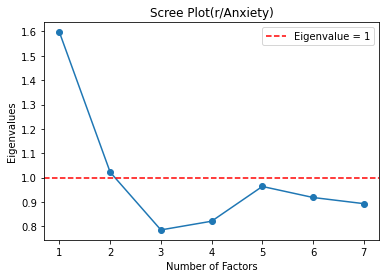 |
| --- | --- |
| 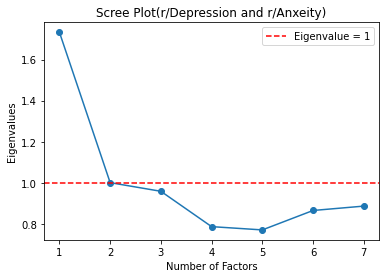 | |

Figure S7. The screen plot of eigenvalues

### Regression Analysis

The OLS regression analysis on the r/Depression and r/Anxiety datasets from 2020 to 2022 showed that the suicide dependent feature had a significant relationship with other independent features such as economic, social, domestic, educational, and substance. Figure S8 displays the result for each subreddit.


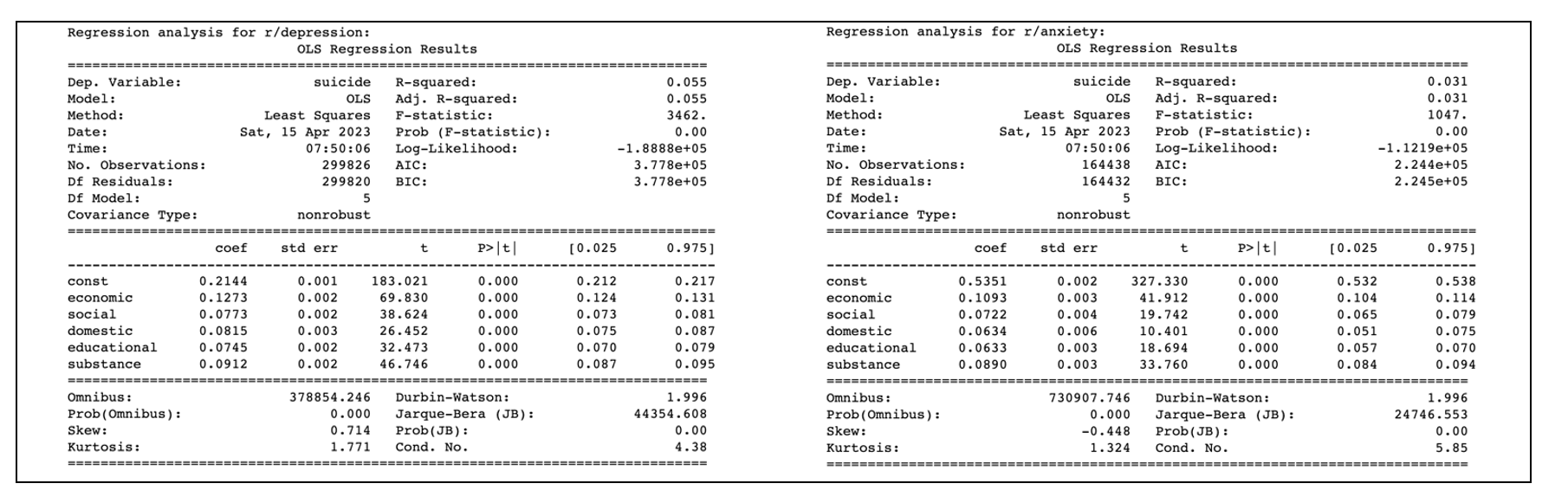


Figure S8. Regression Analysis for the r/Depression and r/Anxiety
